# Structure Modeling Protocols for Protein Multimer and RNA in CASP16 With Enhanced MSAs, Model Ranking, and Deep Learning

**DOI:** 10.1002/prot.70033

**Published:** 2025-08-01

**Authors:** Yuki Kagaya, Tsukasa Nakamura, Jacob Verburgt, Anika Jain, Genki Terashi, Pranav Punuru, Emilia Tugolukova, Joon Hong Park, Anouka Saha, David Huang, Daisuke Kihara

**Affiliations:** ^1^ Department of Biological Sciences Purdue University West Lafayette Indiana USA; ^2^ Department of Computer Science Purdue University West Lafayette Indiana USA; ^3^ Department of Mathematics The University of Texas Austin Texas USA

**Keywords:** CASP, CASP16, kiharalab, model ranking, MSA, protein structure prediction, RNA structure prediction

## Abstract

We present the methods and results of our protein complex and RNA structure predictions at CASP16. Our approach integrated multiple state‐of‐the‐art deep learning models with a consensus‐based scoring method. To enhance the depth of multiple sequence alignments (MSAs), we employed a large metagenomic sequence database. Model ranking was performed with a state‐of‐the‐art consensus ranking method, to which we added more scoring terms. These predictions were further refined manually based on literature evidence. For RNA, we adopted an ensemble approach that incorporated multiple state‐of‐the‐art methods, centered around our NuFold framework. As a result, our KiharaLab group ranked first in protein complex prediction and third in RNA structure prediction. A detailed analysis of targets that significantly differed from those of other groups highlighted both the strengths of our MSA and scoring strategies, as well as areas requiring further improvement.

## 1 Introduction

The three‐dimensional (3D) structure of macromolecules is important for understanding their biological functions and for advancing applications such as precision medicine and drug discovery. While proteins play central roles in cellular functions, RNAs also adopt intricate tertiary structures that are critical for their diverse biological activities, including catalysis, regulation, and molecular recognition. However, experimental structure determination using X‐ray crystallography [1], nuclear magnetic resonance (NMR) [2], and cryo‐electron microscopy (cryo‐EM) [3, 4] is often resource‐intensive and time‐consuming. To complement experimental methodologies, computational structure prediction methods have been actively studied. Experimentally determined structures accumulated in the Protein Data Bank (PDB) [5] over decades have facilitated the development of computational methods for data‐driven structure prediction. The success of these prediction methods has paved the way for a new era in structural biology, significantly advancing our ability to understand and model macromolecular structures.

In protein structure prediction using machine learning, various methods have been proposed to predict different properties of proteins. Earlier methods focused on predicting secondary structures [6, 7, 8], residue‐residue contacts [9, 10, 11, 12, 13], or inter‐residue distances [14, 15, 16]. These predicted properties or constraints have been utilized as constraints to generate 3D structures through physical modeling using methods such as Rosetta [17] and CNS [18]. The first AlphaFold [19], which debuted at CASP13, introduced a convolutional neural network (CNN)‐based architecture using a very deep ResNet to simultaneously predict inter‐residue distances and backbone torsion angles. The predicted results were used as constraints in a gradient descent algorithm to generate 3D structures. AlphaFold's predictions significantly outperformed other methods, demonstrating a major breakthrough in protein structure prediction using machine learning [20]. In CASP14, an advanced version, AlphaFold2 [21], was introduced. Unlike previous models, AlphaFold2 (AF2) implemented two key innovations: A Transformer‐based architecture to extract evolutionary information directly from multiple sequence alignments (MSAs) and a completely new module named Structure Module equipped with Invariant Point Attention (IPA). The most remarkable feature of AF2 is its end‐to‐end architecture, where the network directly predicts all atom coordinates from input MSAs and templates. This not only improved performance but also demonstrated a significant leap in the capabilities of machine learning models for structural biology.

In the previous CASP in 2022, the round 15, no entirely new network architecture that outperformed AF2 emerged. However, several models inspired by the AF2 architecture were introduced [22, 23], along with various techniques aimed at further enhancing the performance of the existing AF2 framework. These included strategies such as enhanced MSA generation [24], deep sampling by running AF2 multiple times [25], as well as selection and ranking techniques that outperformed the standard AF2 scoring function [26].

With the significant advancements in protein structure prediction achieved by AF2, RNA has emerged as the next frontier in structural prediction. RNA structure prediction is more difficult than protein structure prediction because RNA is more flexible, stabilized by standard and non‐standard base interactions, pseudoknots, and long‐range tertiary contacts. Similar to CASP, RNA‐Puzzles [27, 28, 29, 30, 31] provides a benchmarking platform for evaluating RNA structure prediction methods. Since its inception, RNA‐Puzzles has played a crucial role in assessing and advancing RNA structure modeling techniques, including physics‐based modeling [32, 33], homology modeling [34], and, more recently, machine learning‐driven approaches [35, 36, 37, 38, 39]. The RNA structure prediction category was introduced in CASP15 for the first time [40, 41]. In CASP15, traditional approaches leveraging statistical potentials [42] demonstrated greater success than ML‐based methods.

Here, we describe the strategies we employed for protein multimer modeling and RNA structure prediction and the outcome in CASP16. We participated as a human expert group, KiharaLab, and the server group, kiharalab_server, for both categories. As KiharaLab, we submitted all 42 complex targets, while our server submitted 41 targets. For the RNA category, KiharaLab submitted all 44 evaluated targets, while the server submitted predictions for 31 targets. According to the official ranking by the accumulated sum of *Z*‐scores of the assessor's evaluation formula (> −2.0) of model 1, the KiharaLab group was ranked first while the server group was ranked 23rd in the protein multimer category. For the RNA category, rankings were based on the accumulated Z‐scores of the assessor's formula considering all five models with Z > 0. Under this criterion, our KiharaLab group was ranked third and fourth for RNA monomer and multimer rankings, respectively, while our server group was ranked 40th and 30th, respectively. Our approach for protein multimer modeling was to generate hundreds of models with different versions of AF2‐Multimer (AF2M) and database settings, as well as AlphaFold3 (AF3), which was released in early May 2024 at the beginning of CASP16, and rank models based on a consensus ensemble ranking method. We used literature information when available and often performed manual modeling if models from AF2 and AF3 seemed to be unsatisfactory. For RNA, we took an ensemble approach combining a number of the cutting‐edge deep learning methods, including AF3. As with proteins, literature information and manual modeling were also used, but more frequently for RNA due to its difficulty. We describe the methods we employed and showcase examples of both successes and failures and discuss how we can further improve the structure prediction of protein multimers and RNAs.

## 2 Methods

### 2.1 Overview of the Protein Structure Prediction Protocol

**FIGURE 1:**
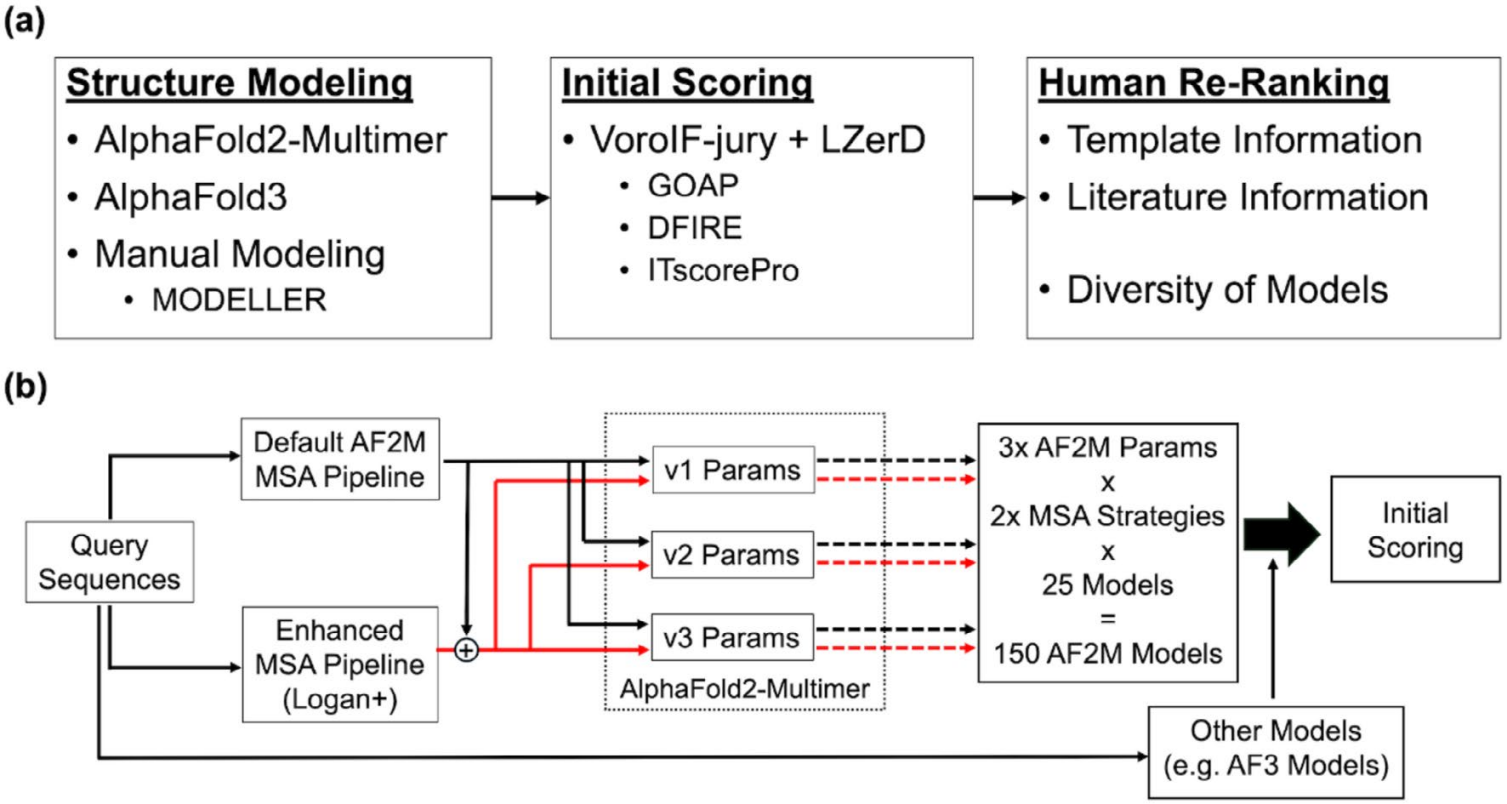
Protein multimer prediction pipeline used in CASP16. (a) Overview of the prediction workflow. The flowchart illustrates the main stages, starting from structure modeling using various methods, followed by initial scoring, and final manual model selection incorporating expert review and curation. (b) Detailed flowchart of the structure modeling stage. Candidate structures were generated in parallel using AF2M and AF3. For AF2M, multiple model weights were combined with two different types of MSAs: A standard MSA and our enhanced MSA with metagenomes. The resulting diverse set of models from both AF2M and AF3 formed the pool for the subsequent scoring and selection stages.

Our human group, KiharaLab, employed an ensemble approach for predicting protein multimer structures in CASP16. The overall workflow of our prediction pipeline is illustrated in Figure 1.

The main structure generation engine we used was AF2M [43]. To generate a diverse set of candidate structures, we used three different official weight releases available at the time of CASP16. Also, we used two different MSAs for each target, a standard MSA generated through commonly used sequence databases and an enhanced MSA that was generated by searching against large metagenome databases (detailed in Section 2.2). Thus, there are 3 × 2 = 6 combinations. Each combination generated 25 structure models; thus, in total, the six variants of AF2M generated 150 models.

In addition, we used the AF3 [44] webserver that became available soon after CASP16 started. The number of AF3 predictions generated per target was adjusted based on perceived target difficulty and the diversity observed among AF3 models. We usually performed one or two runs for seemingly easy targets but increased this to 10 or more runs for particularly challenging cases. Each run of the AF3 webserver produced five models.

As models were generated via different AF versions, we did not compare the models based on their pLDDT confidence scores. Instead, we implemented a multi‐stage ranking and selection pipeline to address this. Initially, models were ranked using a consensus scoring approach, VoroIF‐jury [26], which incorporates 20 variations of ranking derived from structural and knowledge‐based scores (detailed in Section 2.3). We added three additional individual scores to the original set of scores in VoroIF‐jury. This initial ranking was then critically reviewed and curated by team members. During the manual model selection, we used information from relevant literature and ensured structural diversity among the five submitted models. Also, we always included models from both AF2 and AF3. The manual selection has been an important component of our approach in previous CASP and CAPRI rounds [45, 46, 47].

In addition to this standard workflow, target‐specific strategies were applied as necessary. For instance, particularly large complexes were divided into manageable subunits, which were then predicted individually and finally reassembled using tools such as MODELLER [48] or PyMOL. For targets with sparse MSAs, structure‐based homolog searches using Foldseek [49] were employed to potentially enhance the input MSAs. The subsequent sections provide detailed descriptions of the key components of this pipeline, including MSA generation, model ranking, and refinement procedures.

### 2.2 Enhanced MSA Construction With In‐House Metagenome Database

Generating high‐quality multiple sequence alignments (MSAs) is a key for successful structure prediction with AF2M. We updated all databases used for AF2M to the latest versions available at the time. Specifically, we used UniRef30 (2023_03), BFD (latest) [50], UniRef90 (2024_02) [51], UniProt (2024_02) [52], and MGnify NR (2023_02) [53] as the sequence databases. For the template database, we used the Protein Data Bank (PDB) [54] as of April 17, 2024.

Additionally, we constructed an enhanced in‐house MSA database incorporating metagenomic and viral sequence data from Logan contigs (v1.0) [55] and metagenome assemblies from NCBI [56]. To build a sequence database from Logan, we queried two categories: Metagenome (Taxonomy ID: 2787823, unclassified entries) and Virus (Taxonomy ID: 10239, Viruses), downloading all available entries. Since these data give us DNA sequences, we first filtered out sequences shorter than 60 nucleotides and then translated them into protein sequences using Prodigal [57]. Similarly, for the NCBI Assembly data, we searched using Taxonomy ID: 12908 (unclassified sequences), downloaded the sequences, and processed them with Prodigal.

To manage the large volume of sequences, each dataset was clustered using MMseqs2 [58] at a 99% sequence identity threshold. Due to hardware limitations, clustering was performed in batches rather than on the entire dataset at once. Thus, redundancy has not been completely removed, and identical sequences may exist between different batches. The number of sequences in each database is summarized in Table 1.

**TABLE 1:** Statistics of our in‐house metagenome database.

Database name	No of sequences	No of residues	Average length
Logan metagenome	369 561 132 490	25 757 738 226 281	69.7
Logan virus	1 693 109 138	96 970 076 395	57.3
NCBI metagenome	1 401 404 116	235 651 659 966	168.2

We used JackHMMER [59] with the same search parameters as the default settings of AF2 for database search and MSA construction.

### 2.3 Model Ranking With Customized VoroIF‐Jury

As we needed to rank structure models from different settings of AF2M and AF3, an independent model ranking method was necessary. We employed VoroIF‐jury [26] from the Venclovas group because it was successful in CAPRI Rounds 54 and 55 (corresponding to CASP15) as revealed in the CAPRI evaluation meeting held at EMBL Grenoble in February 2024. VoroIF‐jury is a consensus scoring method designed to combine outputs from various scoring functions. Unlike a simple rank aggregation approach, VoroIF‐jury explicitly considers the similarity of predicted interfaces among the top‐ranked structure models selected by different scoring functions. This sophisticated majority voting rewards models representing interfaces that are consistently evaluated favorably by multiple diverse scoring functions.

VoroIF‐jury, which we downloaded from https://github.com/kliment‐olechnovic/ftdmp, implemented four scoring functions developed by the Venclovas lab. In addition, we added three scoring functions, DFIRE [60], GOAP [61], and ITscorePro [62], which we have been using in our group for the LZerD docking protocol [63, 64]. These three scores are statistical potential‐based scoring functions.

### 2.4 Overview of the Prediction Protocol for RNAs

**FIGURE 2:**
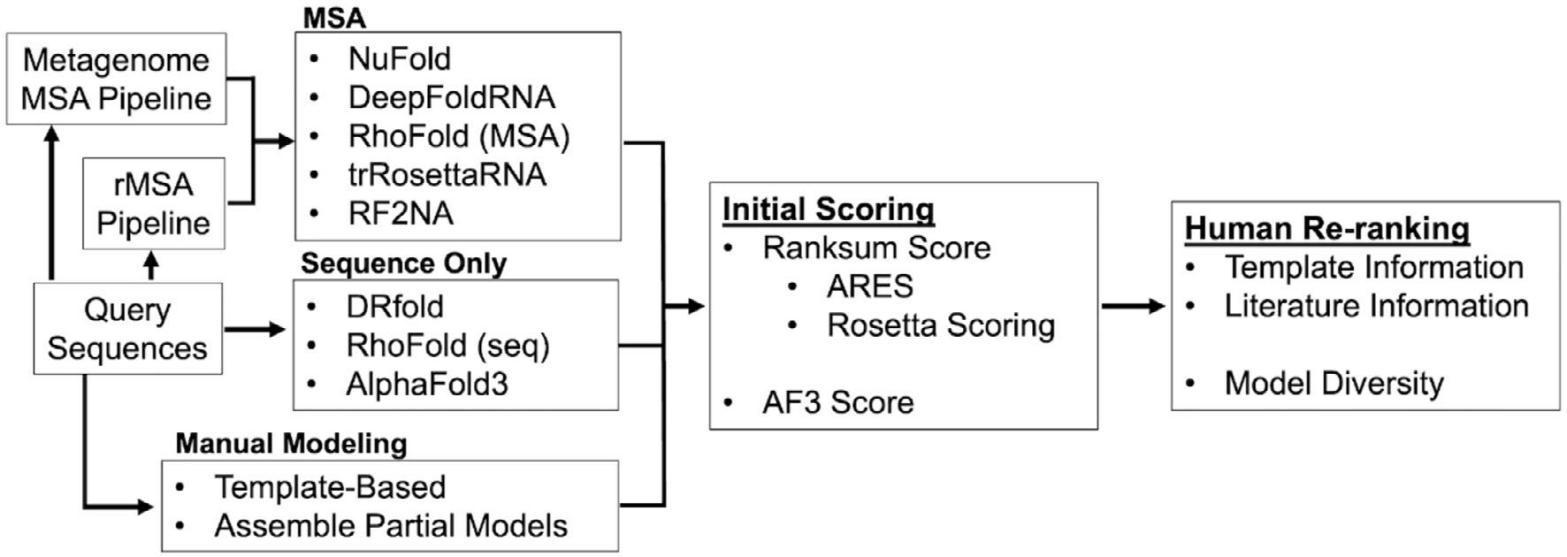
The flowchart of the RNA structure prediction pipeline of KiharaLab. The main workflow starts by MSA construction using rMSA and metagenome databases, structure modeling using seven methods, followed by initial scoring using ranksum, and final manual model selection incorporating expert review and curation.

For RNA structure prediction, we adopted an ensemble approach, shown in Figure 2. We used seven programs, NuFold [35], which is the method developed in our lab, along with six other recent ML‐based methods, namely, DeepFoldRNA [36], RhoFold [37], trRosettaRNA [38], RosettaFold2NA (RF2NA) [22], DRfold [39], and AF3 (web server). MSAs, which are the input for all the methods except for DRfold and the AF3 web server, were computed using the rMSA pipeline [51] with the default parameters. rMSA performs an iterative search against Rfam [65], RNAcentral [66], and NCBI nt [67] using homology search tools such as nhmmer [68], blastn [69], and Infernal [70]. For each RNA target, we also prepared another set of three MSAs using metagenome databases. For the metagenome databases, we used NCBI env_nt, TARA Ocean Metagenome [71], MGnify MAG [53], and all MGnify contigs. Using the search results against the metagenome databases, we constructed the following three MSAs: a raw metagenome MSA (‘meta’), a concatenation of the metagenome MSA with the standard rMSA output (‘Concat’), and a filtered ‘Conca’ MSA where redundant sequences were removed using hhfilter (‘Filtered’). Thus, in total, we had four MSAs for an RNA target, one from rMSA and three with metagenome sequences. We ran each method with four different MSAs, resulting in a total of 140 structure models. In the parentheses, the number of generated models by each method is provided: NuFold (28), DeepFoldRNA (24), RhoFold (4), trRosettaRNA (80), and RF2NA (4). RhoFold and DRfold were also executed using a single sequence mode, which produced one model each for a target. We also used the AF3 webserver to generate at least five models. In total, we produced at least 147 models for a monomer RNA target.

After generating models, we scored them using the Rosetta score [72] and ARES [73], and combined their rankings with the ranksum scoring scheme, which adds up the rankings of individual scores. Similar to protein targets, the final models were examined manually, considering literature and template information, and we ensured diversity of submitted models.

In this pipeline (Figure 2), only RF2NA and AF3 were capable of generating RNA complex structures. However, both methods encountered computational resource limitations when predicting large complexes. In such cases, we predicted the structures of individual subunits or chains using these methods and then assembled them, which sometimes involved manual modeling. Symmetry was applied when the complex exhibited symmetrical architecture. When suitable templates were available, we employed ModeRNA [34]. Manual modeling was primarily performed using UCSF ChimeraX [74].

### 2.5 Evaluation Metrics

Various evaluation metrics have been developed and utilized to assess predicted structures of protein and RNA from multiple perspectives. These include global metrics that evaluate overall structure, such as RMSD, Global Distance Test (GDT) [75], TM‐score [76], as well as local structure assessment scores such as lDDT [77]. For protein complex assessment, metrics focusing on scoring interface such as QS‐score [78] and DockQ [79] have been employed. Tools such as OpenStructure [80], US‐align [81], and structure assessment web server [82] are widely used for calculating these metrics. In the CASP, CAPRI, and CAMEO experiments [83], these metrics have been combined to evaluate predicted structures from various aspects. Furthermore, RNA‐specific evaluation metrics such as interaction network fidelity (INF) [84], which evaluates the accuracy of base‐pairing and stacking interactions that are crucial for RNA structure stability, have been introduced. The INF score has been used in RNA‐puzzles experiments [27, 28, 29, 30, 31] along with RMSD, TM‐score, and lDDT. Inspired by this INF score, a more generalized version of the INF score is developed to evaluate the interface of macromolecular complexes [85].

In this study, we used DockQ as the score for protein complex prediction. DockQ is a composite metric that integrates interface RMSD (i‐RMSD), ligand RMSD (l‐RMSD), and the fraction of native contacts (Fnat), yielding a single score ranging from 0 to 1. Higher DockQ values indicate more accurate predictions of interaction interface.

For RNA monomer evaluation, we used TM‐score, a global metric for assessing overall structural similarity. TM‐score ranges from 0 to 1, with scores above 0.5 generally considered to indicate correct fold. As TM‐score is length‐normalized, it enables consistent evaluation across RNAs of varying sizes.

## 3 Results and Discussion

### 3.1 Overall Results and Statistics of Protein Multimer Targets

**FIGURE 3:**
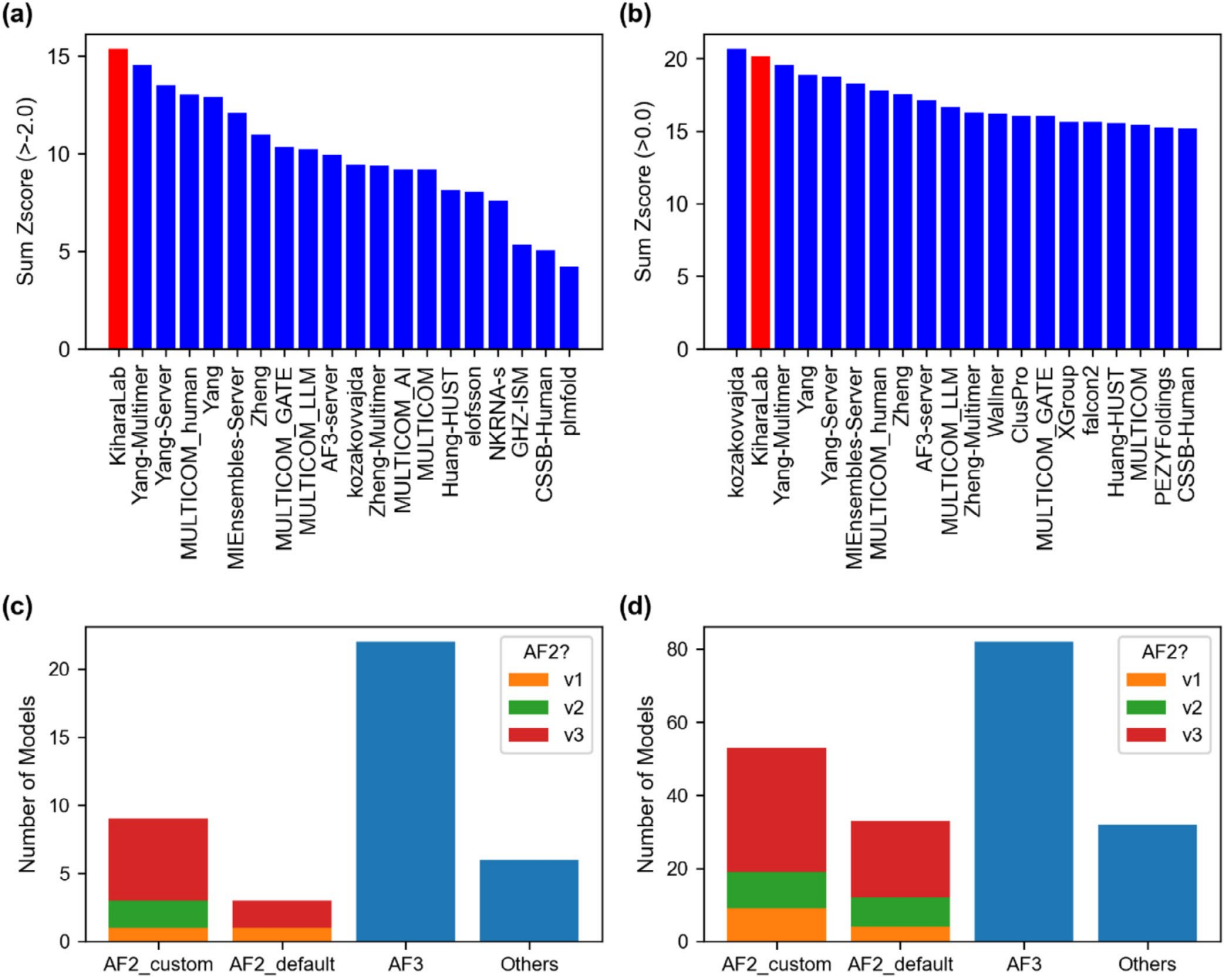
Overall result of protein multimer modeling of KiharaLab human group in CASP16. (a, b) Group rankings in the multimer category. The top 20 teams are shown. Our group (KiharaLab) is shown in a red bar. A total of 37 targets were evaluated. The ranking of the top‐1 models is shown. (a) Ranking by Sum Zscore (> −2.0). (b) Ranking by Sum Zscore (> 0.0). (c) Breakdown of source of top 1 models. We submitted models for 40 multimer targets. (c) Source of all five submitted models.

In the Multimer category, a total of 86 prediction groups, including both server and human groups, submitted at least one prediction and were ranked. According to the official rankings on the CASP16 website (https://predictioncenter.org/casp16/zscores_multimer.cgi), our human group, KiharaLab (TS294), achieved the top position in the top 1 ranking when targets with sumZ > −2.0 were considered, and ranked second when targets with sumZ > 0.0 were considered (Figure 3a,b). This strong performance indicates that our prediction protocol consistently produced high‐quality predictions across a wide range of targets. When all five submitted models were considered (top 5 ranking), our group ranked fourth for sumZ > −2.0 and sixth for sumZ > 0.0. The difference between the top 1 and top 5 rankings suggests that our model selection strategy was effective in identifying the best prediction among the five submitted models.

In Figure 3c,d we show the breakdown of the sources of structure models we submitted as our top 1 models and all five models, respectively. Our selection strategy ensured that each target included at least one AF2‐based model and one AF3‐derived model, and models with manual intervention, for example, models that used a MSA with sequences collected by Foldseek, if any. When top 1 models were considered (Figure 3c), 22 out of 40 targets (55.0%) were from AF3. AF2‐based models accounted for 12 (30.0%), with models from the v3 parameters being the most selected (8 targets). When all five models were considered (Figure 3d), 82 out of 200 models (41.0%) were from AF3. AF2‐based models accounted for 86 models (43.0%) with models from the v3 parameters being the most selected (55 models). Thus, overall, AF3 models were most frequently selected as the top 1 model, while the set of five submitted models included models from more diverse sources.

Models categorized as “other” sources comprised 15.0% of the top 1 submissions and 16.0% of the top 5 submissions. These models originated from various approaches. For very large complexes where modeling the entire structure was infeasible, subsets of interacting chains were modeled using AF2 or AF3 and subsequently assembled using tools such as MODELLER or PyMOL. When available, templates or information from the literature were also incorporated. Additionally, we examined models submitted by other groups during Phase 0. These models were ranked using our initial scoring scheme and further evaluated through manual inspection.

### 3.2 Effect of the MSA Size on Protein Assembly Modeling Performance

**FIGURE 4:**
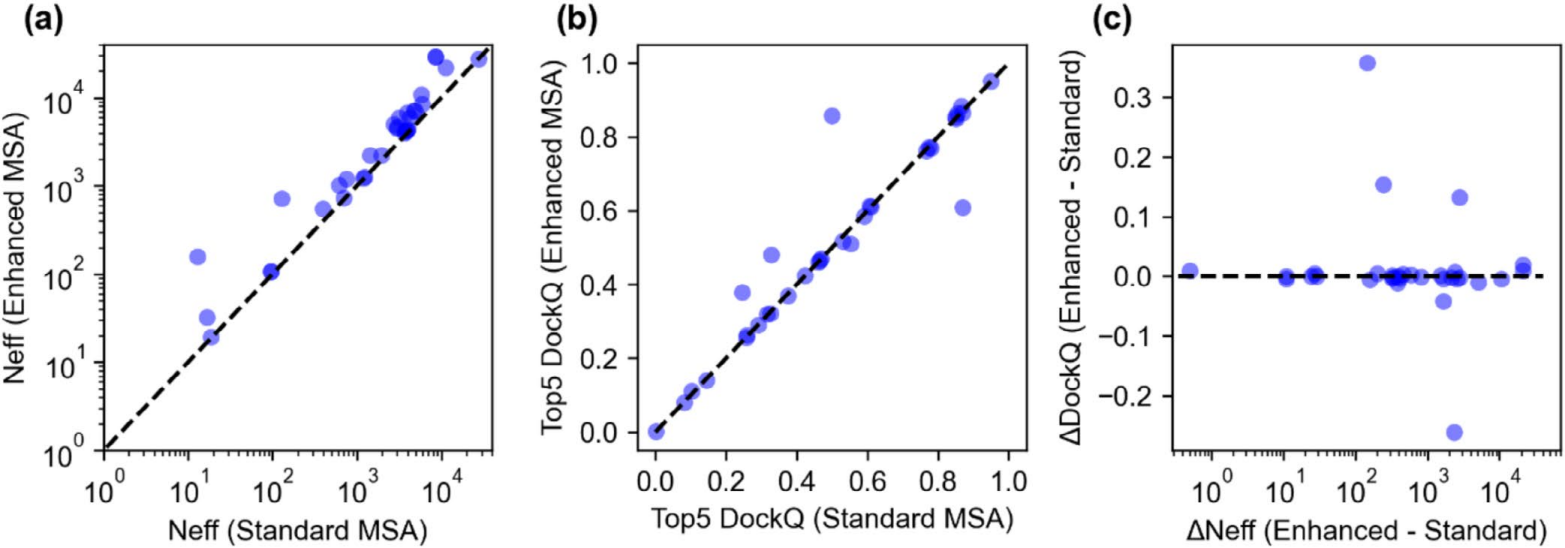
The effect of Neff of MSAs to DockQ. (a) The number of effective counts (Neff) for the standard MSAs in AF2 and the enhanced MSAs from the Logan metagenome data. As enhanced MSAs were always added to the standard MSAs, Neff remains the same or increased by the metagenomes in all cases. Both axes are in a log scale. (b) Difference in structure prediction performance with the standard MSAs and the enhanced MSAs. The structures were predicted using AF2 v3, and only the MSAs were modified. The top 5 structures were obtained with the standard ranking metric in AF2 and reported the highest DockQ among them. (c) Relationship between change in Neff (ΔNeff, x‐axis) and change in DockQ (ΔDockQ, y‐axis). Changes on both axes are calculated by subtracting standard from Enhanced; thus, a positive value in ΔDockQ indicates improvement.

In Figure 4, we analyzed the depth of MSAs relative to the modeling performance of the protein multimer prediction. We measured MSA depth using Neff, which represents the effective number of unique sequences after redundancy removal. Neff is calculated using NEFFy [86] with a sequence similarity cutoff of 0.8. For the protein assembly category, we had two MSAs for each target, one from the standard database and the other from an in‐house metagenome database constructed from the Logan contigs.

Figure 4a shows Neff of the enhanced MSAs from Logan relative to that of the default AF2 MSA. As shown, the enhanced MSA consistently increased Neff compared to the default AF2 MSA. On average, Neff increased by 2490.1 (1.66‐fold) by using the enhanced MSAs. The most significant improvement was observed for target T1235, where the original MSA had a Neff of 13.0, while our enhanced MSA increased it to 157.7. For this target, this increase in Neff led to a DockQ improvement from 0.500 to 0.857.

In Figure 4b, we examined the modeling performance in terms of DockQ with the enhanced MSAs and the default AF2 MSAs. As shown, DockQ stayed almost the same for most of the targets, but a substantial increase in DockQ was observed for three targets. These are targets H1236, H1258, and T1235, where Neff of the enhanced MSA increased by 1.12‐fold (from 1966.2 to 2208.0), 1.70‐fold (from 3966.2 to 6768.0), and 12.1‐fold (from 13.0 to 157.7), respectively. On the other hand, for the target T1259, DockQ decreased from 0.870 to 0.609. In this case, Neff of the enhanced MSA increased by 1.87‐fold (from 2699.7 to 5036.0). There was no clear correlation observed between the increase in Neff and improvement in DockQ (Figure 4c). This lack of correlation may be attributed to several factors, including the diminishing benefit of deeper MSAs beyond a certain threshold and the potential introduction of noise in the enhanced MSAs.

### 3.3 Revising Model Ranking for Protein Multimer and RNA Targets

**FIGURE 5:**
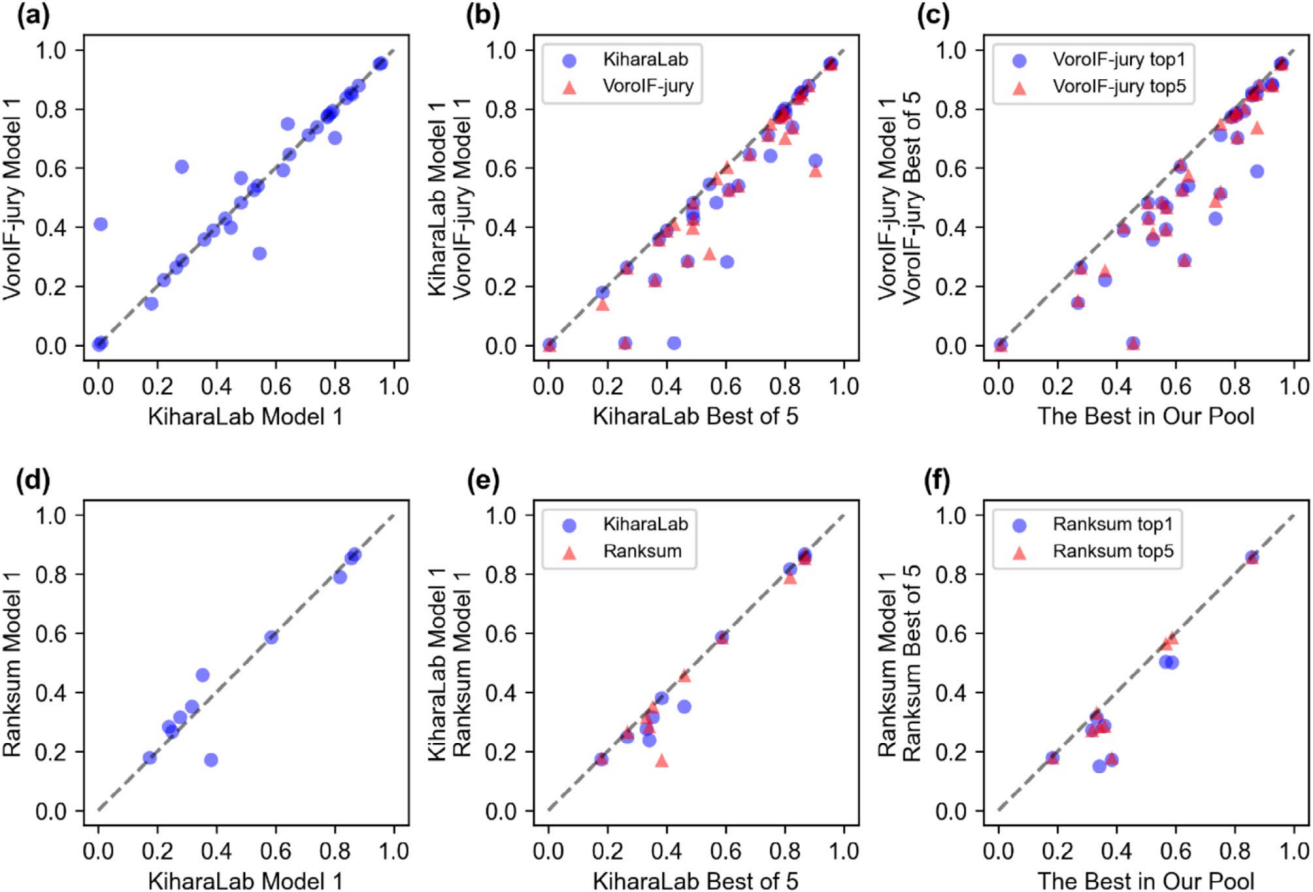
Evaluation of scoring schemes. Panels a‐c show score rankings of protein multimer models. Evaluation of models was performed with DockQ score. (a) Comparison between KiharaLab Model 1 and the model with the best VoroIF‐jury score among the five models submitted by KiharaLab. (b) Comparison among the best model out of KiharaLab's five submissions, KiharaLab Model 1 (blue circles), and VoroIF‐jury Model 1 (red triangles). (c) Comparison between the best DockQ model in our entire model pool and the top‐1 (blue circles) and top‐5 (red triangles) models selected by VoroIF‐jury. (d–f) The analysis of RNA monomers. Evaluation of models was performed with TM‐score. (d) Comparison between KiharaLab Model 1 and the model with the best ranksum score among the five models submitted by KiharaLab. (e) Comparison among the best model out of KiharaLab's five submissions, KiharaLab Model 1 (blue circles), and ranksum Model 1 (red triangles). (f) Comparison between the best TM‐score model in our entire model pool and the top‐1 (blue circles) and top‐5 (red triangles) models selected by ranksum.

In this section, we discuss model rankings for protein multimer and RNA targets. Figure 5a–c illustrate the model rankings generated by our modified VoroIF‐jury for protein multimer targets. In Figure 5a, we compare the DockQ scores of the top‐ranked model according to VoroIF‐jury with those of Model 1, the model actually submitted by our human group, for each target. For the majority of targets (19 out of 33), Model 1 matched the model with the highest VoroIF‐jury score. However, in 12 cases, the selected models differed. Among these, four showed a DockQ difference greater than 0.1. In three of those four cases, the model with the highest VoroIF‐jury score outperformed the manually selected Model 1 in terms of DockQ. In the remaining case, the manual selection yielded a higher DockQ. The three targets where the VoroIF‐jury‐selected models had higher DockQ scores were H1222, H1236, and T1249v1, with respective DockQ comparisons of 0.750 versus 0.641, 0.604 versus 0.282, and 0.410 versus 0.008 for the VoroIF‐jury‐ranked models and the human‐selected Model 1. For the case of H1222, the overall peptide arrangement was visually very similar between the top human‐selected model and the VoroIF‐jury top‐ranked model. However, the human‐selected model featured a twisted and more separated conformation of the CDR3 loop of the heavy chain, which differed from the VoroIF‐jury model and was the wrong conformation. In H1236, the human Model 1 selected a structure where the trimer of the A chain complex deeply penetrated the ring formed by the hexamer of the B chain. Many decoys, including all the AF3 structures, had similar structures, which led to its selection by the human group. In contrast, the VoroIF‐jury top‐scoring structure had a markedly different conformation that lacked this penetrating part and the hexamer ring was flipped. In the case of T1249v1, the human group manually selected a model with a more open conformation compared to the T1249v2 “closed” conformation. However, by comparing with the native structures, the domain exhibiting open/close movement turned out to be different from what we initially assumed, and the conformation change was smaller than that. The human‐selected “open” model placed the domains in a widely separated position, resulting in a significant reduction in the DockQ score.

Conversely, for H1223, the human group selected a model with a DockQ of 0.545 as Model 1, whereas the top VoroIF‐jury model had a score of 0.311. In this case, both models failed to correctly dock the peptide. However, the human‐selected Model 1 favored a larger docking interface between the antibody and peptide, which allowed it to partially capture the correct docking residues of a peptide.

In Figure 5b, we evaluated whether the models selected by VoroIF‐jury and by human judgment were indeed the best among the five submitted models. If a selected model was the best structure among the five, its corresponding point appears on the diagonal in the plot. For eight out of 33 targets, the top‐ranked models by VoroIF‐jury and human selection matched the best model among the five submitted. The average DockQ scores for the best of the five models, the top VoroIF‐jury selection, and the top human selection were 0.618, 0.562, and 0.548, respectively. There were six and eight cases where the top‐ranked model by VoroIF‐jury and human selection, respectively, deviated from the best model by more than 0.1 in DockQ. One such example is target H1233, where Model 3 from the human submission achieved a DockQ of 0.903, yet neither the human nor VoroIF‐jury selection identified it as the top model. Another example is target T1249v1, where Model 4 from the human submission had a DockQ of 0.424, but the human Top 1 selection was entirely incorrect, with a DockQ of 0.008. For this target, VoroIF‐jury's Top 1 model achieved a DockQ of 0.410. Therefore, based on Figure 5a,b, the overall results would have been slightly better, retrospectively, if we had relied entirely on VoroIF‐jury for ranking the five submitted models. This is somewhat understandable, as we occasionally prioritized a model as Top 1 when it had been built with special efforts, such as manual modeling or innovative ideas, which we believed had the potential for a substantial performance gain over submissions from other prediction teams.

In Figure 5c we further analyzed the ability of VoroIF‐jury to identify high‐quality structures from the full pool of at least 155 structure models. When the top five models were considered, VoroIF‐jury was able to select a model within 0.1 DockQ of the best model for 66.7% of the targets (22 out of 33). For three targets, it successfully selected the best model from the pool. Thus, although there is still room for improvement, the scoring performed reasonably well overall.

Next, we conducted a similar evaluation for RNA scoring using Figure 5d–f. We evaluated only 11 RNA‐only monomer targets. RNA multimers, protein–RNA complexes, and protein–ligand complexes were excluded from this analysis, as no ranking method was applied to them. For RNA targets, we used a ranksum score that combined the Rosetta energy score and ARES scores [73]. Figure 5d compares the TM‐score of the submitted Model 1 with that of the model having the highest ranksum score among the five submitted structures. In six cases (54.5%), the model selected by ranksum had a higher TM‐score than the human‐selected Model 1. The average TM‐score for the top‐ranked models by ranksum and human selection was 0.466 and 0.465, respectively. In Figure 5e, we compared the ranksum and human selections to the best‐performing model among the five submitted. In five cases for ranksum and three cases for human, the best model was correctly selected. Notably, when a high‐quality model (e.g., a TM‐score of over 0.5) was present in the pool, both methods generally succeeded in identifying it. However, based on Figure 5d,e, ranksum would have yielded a slightly higher overall performance compared to human selection.

Figure 5f investigates ranksum's selections from model pools that varied across targets (ranging from 39 to 222 models), with longer targets generally associated with smaller pools due to time and hardware constraints. When the top five choices were considered, ranksum selected the best model in the pool for four targets out of nine (44.4%). In addition, for eight cases, the best among the five choices by ranksum was within 0.1 TM‐score of the best model. Ranking RNA monomer models was generally more straightforward than ranking protein multimer models, due to the overall lower quality of the RNA monomer model pool, which made high‐quality models more distinguishable. It is important to note that this observation is specifically relevant to RNA monomer modeling, underscoring the inherent difficulty of accurately predicting even single‐chain RNA structures in comparison to protein modeling. In particular, for long RNA sequences, all models showed obvious structural flaws, except for AF3 models.

### 3.4 Comparison of Our Human Group With Automated Servers

**FIGURE 6:**
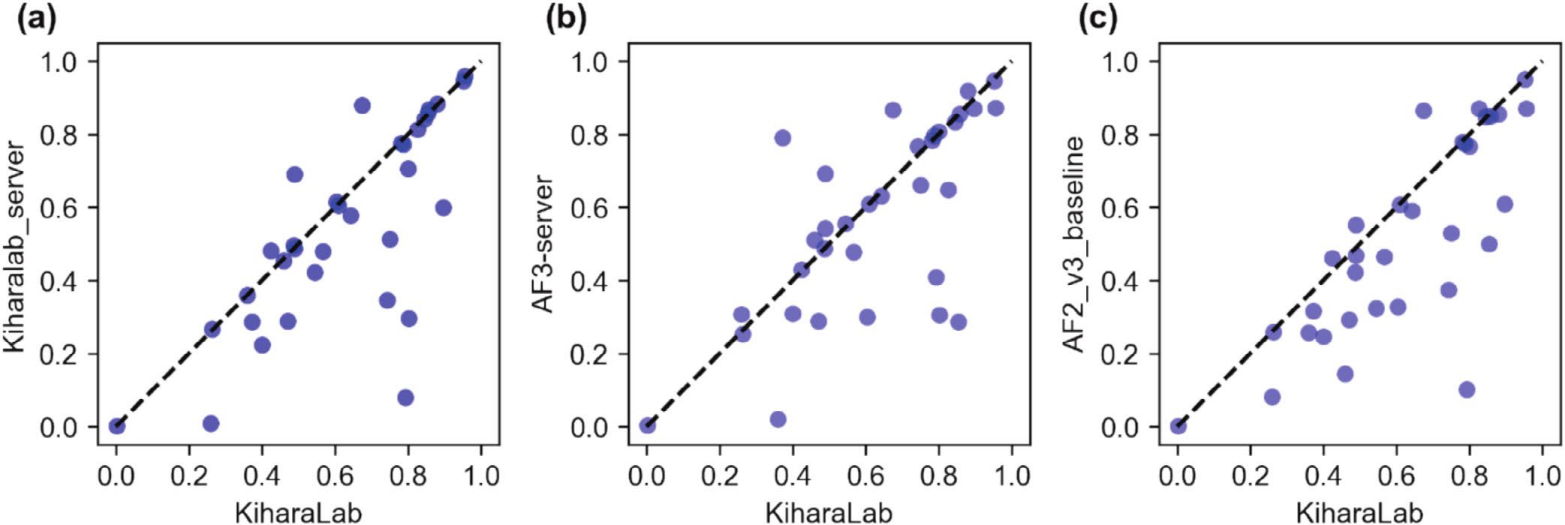
Comparison between our human group and server methods. The values are Top5 DockQ. The x‐axis are submissions from KiharaLab (TS294) compared with (a) Kiharalab_server (TS267); (b) AF3‐server (TS304); (c) AF2 v3 baseline.

In Figure 6 we compared the modeling performance of our human group's submission with our server group, Kiharalab_server (TS267), the AF3 server, and AF2 with version 3 (the latest) weights. The DockQ score is used for this comparison.

Figure 6a is the comparison with our server. The server group employed an automated procedure where it runs AF2 with the three parameter sets, ver1, 2, and 3, using a standard MSA followed by model ranking using VoroIF‐jury. Therefore, compared to the server group, differences in the human groups are that the human group used enhanced MSAs, used AF3, and human intervention in model inspection and ranking. As shown in the plot, the human group had substantially better DockQ scores over the server group. The average DockQ of the Top 5 for the human group was 0.626, whereas it was 0.542 for the server group. Out of 33 targets in total, the human group's DockQ score was higher for 21 targets.

When compared with the AF3‐server group (TS304), which ran AF3 with its default parameters (Figure 6b), our human group achieved a higher DockQ score on 17 targets (51.5%) when top five submissions were considered. The average DockQ of the AF3‐server group was 0.571, only slightly higher (by 0.029) than that of our server group. The largest difference in DockQ was observed for target T1235 (A6), where the human group achieved a DockQ of 0.854, while the AF3‐server group reached only 0.287. This target is gp30 (also known as turret base protein), TBP from Haloferax tailed virus 1 (HFTV1). It is known that viral protein structures are generally more challenging to predict with AlphaFold, and this target might be one such case. The high‐scoring model for this target by our human group was generated by AF2M with v3 weights and an enhanced MSA. The effectiveness of the enhanced MSA in this case may stem from the fact that it was constructed by searching the metagenomic databases that include viral sequences (Table 1).

We also compared our results to the AF2M baseline with v3 weights (Figure 6c), which represents the performance of the latest AF2M without modification. The average DockQ score for this method was 0.511. Our human group outperformed this baseline by more than 0.1 DockQ on 14 targets. Since this baseline is similar to our server method, it also failed to predict the correct structure for target H1245, achieving a DockQ score of only 0.102.

### 3.5 RNA Structure Prediction Results

In CASP16, a total of 39 targets involving RNA were released. Among them, only 12 (30.1%) were RNA monomers. The remaining 27 targets included 12 protein‐RNA complexes, 11 RNA–RNA complexes, and four RNA‐ligand complex targets. Most available RNA structure prediction methods are primarily designed for monomer RNA modeling. These include our own tool, NuFold, as well as other recent methods such as DeepFoldRNA, RhoFold, trRosettaRNA, and DRfold. In contrast, RosettaFold2NA and AF3 are capable of predicting RNA‐containing multimeric structures, such as RNA–RNA and RNA‐protein complexes. Large RNA targets were also challenging for these methods. For larger targets, many modeling attempts failed due to excessive memory requirements or computational time constraints. AF3 generally demonstrated strong performance across various sizes of RNA targets. However, for homomeric RNA complexes, AF3 frequently generated physically implausible structures in which all chains collapsed into a single, overlapping region. Although such errors were easy to identify, addressing and preventing them remain challenging.

As described in the Methods section, we used multiple RNA monomer models and ranksum score for initial ranking. For targets that were not RNA monomers or were relatively large RNAs, the submitted models were primarily generated using AF3. While AF3 produced largely consistent secondary structural elements, such as stems, the global structures often differed. For some targets, magnesium or potassium ions were known to play important structural roles, and in such cases, these ions were included in the input to AF3 with various concentrations. The presence of these ions sometimes resulted in different RNA conformations, although in some instances, the structures remained unchanged. When a target was too large to be directly modeled with AF3, we attempted to predict the structure of the largest possible number of chains using AF3. Based on these predicted structures and observations, we manually constructed and applied symmetry operations, considering the interaction patterns observed in the smaller stoichiometry predictions, to construct models with the designated stoichiometry. ChimeraX was primarily used for this purpose. In particular, structures with severe clashes, or those resulting from these manual operations, were relaxed using QRNAS [87] or Amber relaxation [88].

If it was only AF3 that produced meaningful structures, the AF3 scores were used for initial ranking. In cases where literature provided a preference for a particular structure, we took that into account and overrode the AF3 rankings accordingly. For difficult targets lacking sufficient literature information, AF3 tended to generate a wide range of structural variants, and we selected five models to cover this diversity. When manual modeling was performed, we typically did not generate more than five models. These were then ranked visually and submitted. This visual assessment primarily checked for the absence of unrealistic structural features, such as severe clashes or unphysical chain topologies, and ensured that interaction interfaces were not unreasonably small. We also paid attention to the formation of long‐range interactions, such as pseudoknots, which are generally challenging to predict, and models with such features were favored. Furthermore, when multiple distinct conformations were present among the generated models, we selected a diverse set to represent this structural variability in our submission.

**FIGURE 7:**
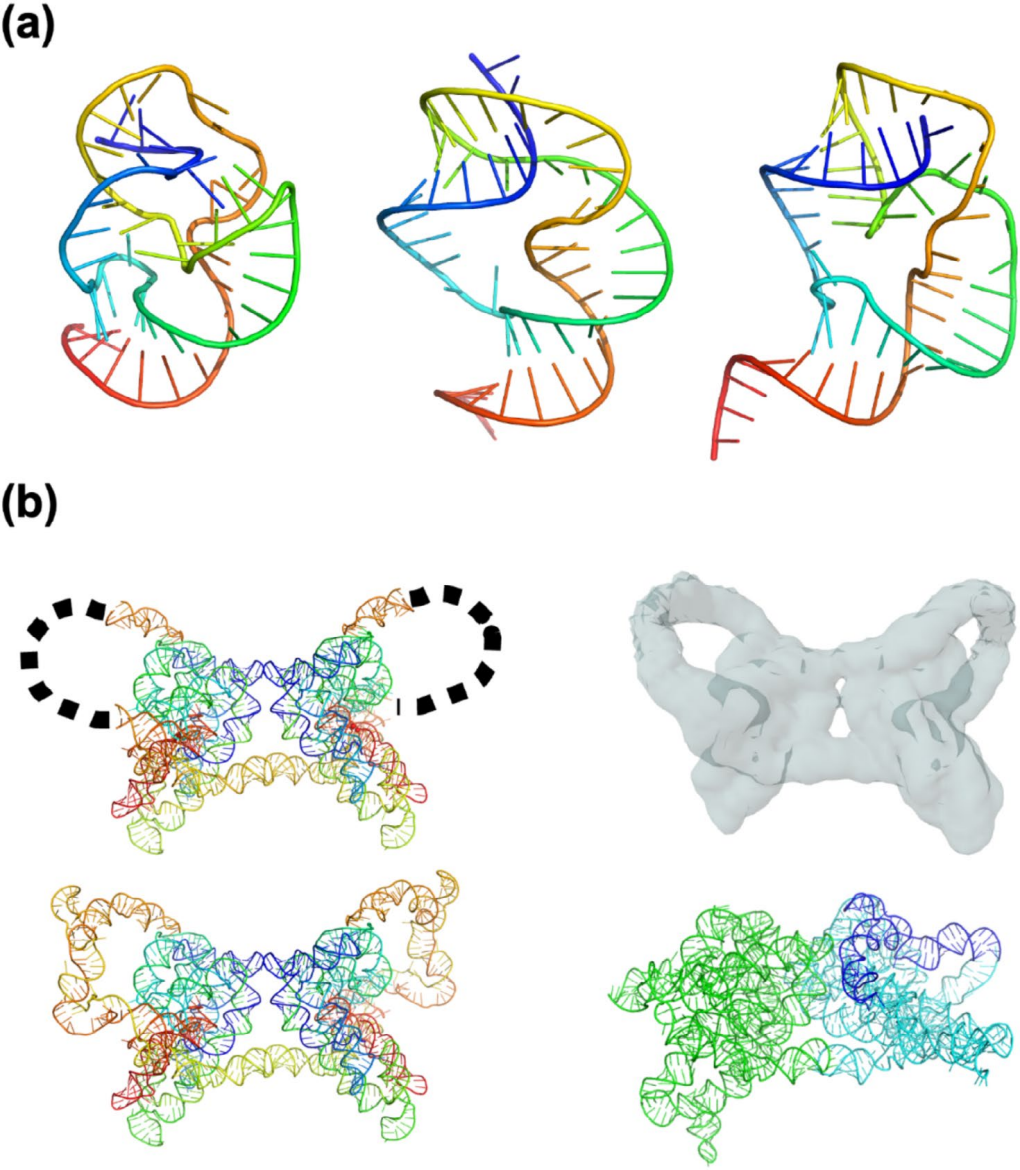
Examples of RNA structure models. (a) Target R1205. Left: Native structure (figure provided by the CASP organizers). Middle: Our model 2 (R1205TS294_2) predicted by NuFold. Right: Our best model, model 3 (R1205TS294_3) predicted by AF3. (b) A successful manual modeling for R1290. Left top: The template structure we used (PDB ID: 8K0P). Right top: Cryo‐EM density map of 8K0P (EMD‐36776). Left bottom: Our best model (R1290TS294_1o). Right bottom: Predicted structure from AF3. The missing loop region in the template structure was colored in blue.

We ran our NuFold for 11 single‐chain RNA targets. A notable example highlighting the potential of our NuFold method was target R1205, an exoribonuclease‐resistant RNA (xrRNA). For this target, both NuFold and AF3 successfully captured the key topological feature characteristic of xrRNAs: the threading of the 5′ end through a loop to form a specific pseudoknot structure (Figure 7a). Although the overall predicted structures did not perfectly match the native conformation—achieving TM‐scores of 0.353 (NuFold) and 0.338 (AF3)—correctly modeling this intricate pseudoknot topology marked a significant accomplishment, particularly in comparison to methods that failed to capture this feature. Our model achieved the fourth‐highest TM‐score among all submissions by participating teams. Compared to Model 4 of the top‐performing submission by GuangzhouRNA‐meta, which scored a TM‐score of 0.360, our model was only 0.007 lower.

A notable example of our successful manual modeling was the target R1290, a homodimer RNA, shown in Figure 7b. For this target, PDB ID: 8K0P was available as a structural template, which had 99% sequence identity to the target. However, this template structure has a large missing loop in the middle of the structure. The AF3 prediction was unable to generate a structure in which the entire RNA structure matched the template well enough. Also, the tip of the loop in the template contacts the main part of the structure, but the AF3 structure completely missed this part. However, the loop part of this AF3 structure was roughly consistent with the secondary structure features reported in the original 8K0P study [89]. Therefore, the loop structure created by AF3 and the template structure were combined using ModeRNA [34]. In this way, a model was created that combined the strength of both the already known template structure and the relatively correct predicted loop structure. By looking at the cryo‐EM density map, EMD‐36776, used to determine the structure of 8K0P, the rough feature of this loop could be seen at a lower contour level, and it was found to have the same topology as the predicted structure. Our submitted model 1 achieved a TM‐score of 0.996, a near‐perfect score.

### 3.6 What Went Well?

In this section, we present three cases where our structural modeling performed particularly well. Our modeling pipeline particularly excelled in targets involving viral assemblies, an area that is often challenging due to limited evolutionary information or high conformational variability. We attribute our success to two main factors. The first is the use of enhanced MSAs, especially incorporating metagenomic sequences, which improved the quality of inputs to AF2. The second is a robust model selection strategy with VoroIF‐jury and carefully evaluating candidate structures through both automated scoring and manual inspection.

**FIGURE 8:**
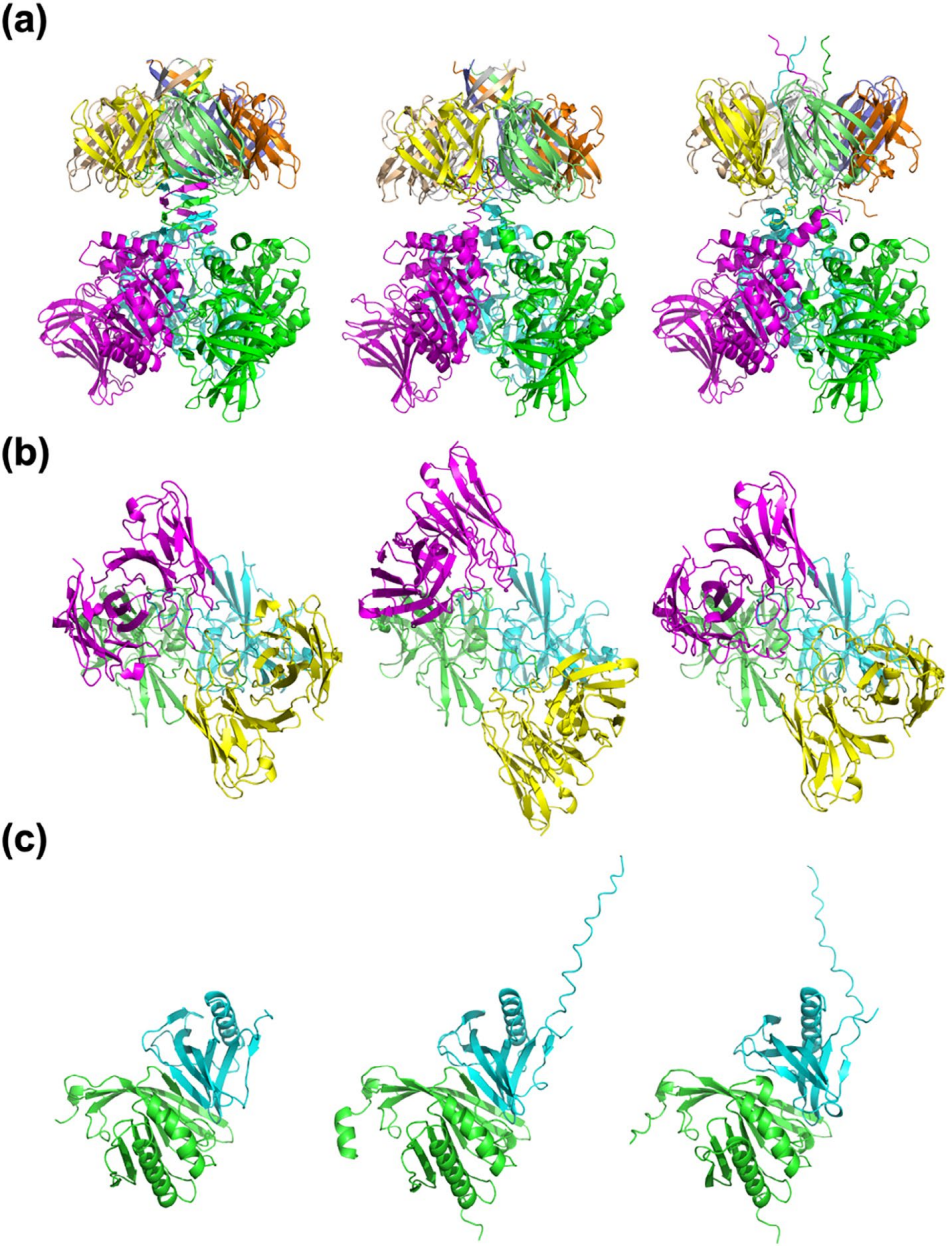
Successful protein multimer structure prediction examples. (a) Prediction results of target H1236. Left: Native structure (PDB ID: 8QPQ). Middle: Our best model (H1236TS294_3) DockQ: 0.604; RMSD: 6.2 Å. Right: AF3‐server best model (H1236TS304_1). DockQ: 0.301; RMSD: 15.6 Å. (b) Prediction results of target H1232. Left: Native structure (PDB ID: 9CN2). Middle: Our best model (H1232TS294_4). DockQ: 0.567; RMSD: 11.1 Å. Right: The best model of all submissions by smg_ulaval (H1232TS397_2). DockQ: 0.680; RMSD: 3.5 Å. (c) Prediction results of target H1245. Left: Native structure (CASP organizer provided). Middle: Our best model (H1245TS294_1). DockQ: 0.793; RMSD: 1.5 Å. Right: The AF3 model we submitted (H1245TS294_5). DockQ: 0.455; RMSD: 3.1 Å.

The notable success for our group was achieved for the multimer target H1236, representing the C1 turret‐capsid interface with its A3B6 stoichiometry from the Haloferax tailed virus 1 (PDB: 8QPQ). Our submitted prediction, Model 3, achieved a high DockQ of 0.604 and an RMSD of 6.166 Å against the native structure. This performance was particularly strong, although the numerically top‐ranked model overall was submitted by the automated kiharalab_server, achieving a DockQ of 0.615 and RMSD of 5.876 Å. Our best models significantly outperformed models from other groups; for comparison, the best submission from another group, MassiveFold model 2, achieved a DockQ of 0.379, and the AF3‐server model reached a DockQ of 0.301. Both of these latter models exhibited much higher RMSDs of over 15 Å. Visual inspection revealed a key structural difference contributing to the higher accuracy of our model compared to these other predictions. Our Model 3 (Figure 8a) correctly positioned the ring‐like B6 hexamer assembly relative to the A3 trimer. In contrast, both the MassiveFold and AF3‐server models featured the B6 hexamer flipped by approximately 180°, leading to a gross misplacement of the subunit and a failure to capture its N‐terminal beta‐barrel fold. This misorientation explains their high RMSDs.

Our successful Model 3 was generated using AF2 with enhanced Multiple Sequence Alignments (MSAs) as input. We hypothesize that the improved MSA quality was a critical factor in achieving the correct quaternary arrangement. Viral proteins can be challenging for MSA generation due to sequence divergence or scarcity of homologs. For H1236, our enhanced pipeline substantially increased the number of effective sequences, Neff, for the B6 component from a baseline of 13.0 to 157.7 by incorporating metagenomic sequences. This richer evolutionary information likely provided crucial constraints for AF2 to correctly fold and orient the B6 subunit, an aspect where other methods using potentially shallower MSAs struggled. Regarding the A3 component, a partial template (PDB: 5JMU) was identified, covering the larger domains but lacking the connecting domain, a tube‐like extension that inserts into the B6 ring and forms the primary contact between the subunits. Accurately modeling the beta‐sheet structure within this non‐templated connecting domain proved challenging, with both our model and the AF3‐server model predicting erroneous helix structures in this region.

The second case, H1232, involved the homodimer human astrovirus 2 capsid spike protein in complex with an antibody (Figure 8b). While we could confidently predict the capsid protein structure, accurately modeling the antibody epitope remained a significant challenge. Both AF2 and AF3 generated diverse complex structures, with antibodies binding to various antigenic surfaces. Consequently, we performed an extensive sampling of AF3‐generated complexes, selecting five structures based on scoring and visual inspection. Our best structure, Model 4, achieved a DockQ of 0.567 and was generated by AF3. This model ranked second overall among all submitted structures. Notably, one submission from smg_ulaval (TS397) achieved a DockQ of 0.680, closely matching the native structure and accurately identifying the epitope.

The third case, H1245, was a heterodimer target described as a toxin/antitoxin complex, shown in Figure 8c. Our best submission, Model 1, was selected based on scoring from the combined structure model pool, including our own models and all phase 0 submissions, and was submitted by NKRNA‐s (TS028). This model achieved a high DockQ of 0.793, making it the 10th best structure among model 1 submissions overall. In terms of DockQ, Yang‐Multimer achieved 0.830, as the best performance in all model 1 submissions. Our best structure was also independently selected by the three Yang group methods. A key challenge in this target was the prediction of the beta‐sheet on the dimer interface. AF3 struggled with this aspect, failing to correctly predict the interfacial beta‐sheet conformation. Among our submissions, Model 5, which was generated by AF3, was the second best, achieving a DockQ of 0.455 but ranking 74th among all submitted models. The results showed that our modeling approach was unable to generate the best structure for this target. However, it revealed that our scoring method was effective in identifying the most accurate structure, highlighting its utility.

Regarding RNA structure modeling, we found that NuFold sometimes performed well for smaller RNAs, even outperforming AF3 (targets R1205). Supported by our scoring function using ranksum, we were able to select high‐quality models in these cases. The majority of RNA targets in this CASP round were either a part of RNA–protein complexes or RNA–RNA complexes, for which other modeling tools we employed generally did not work or did not perform well, making the use of AF3 indispensable. In all cases, literature research was critical for RNA targets, which gave us information about secondary structure or interaction patterns. The integration of published structural information into our modeling and model selection processes was necessary, and the success we achieved in RNA modeling may be attributed to our expert ability to effectively incorporate this information.

### 3.7 What Went Wrong?

Our modeling pipeline did not work well for targets with nanobody and antibody. It is known that modeling complexes with antibodies and antigens is challenging because they do not share coevolutionary signals, and antibodies typically interact with their targets through small and highly selective interfaces [90, 91]. In our predictions, our model 1 was unable to recognize the correct epitopes on any of the eight targets with antibody or nanobody. No meaningful improvement was observed for all targets except H1233 when considering all five submitted models.

**FIGURE 9:**
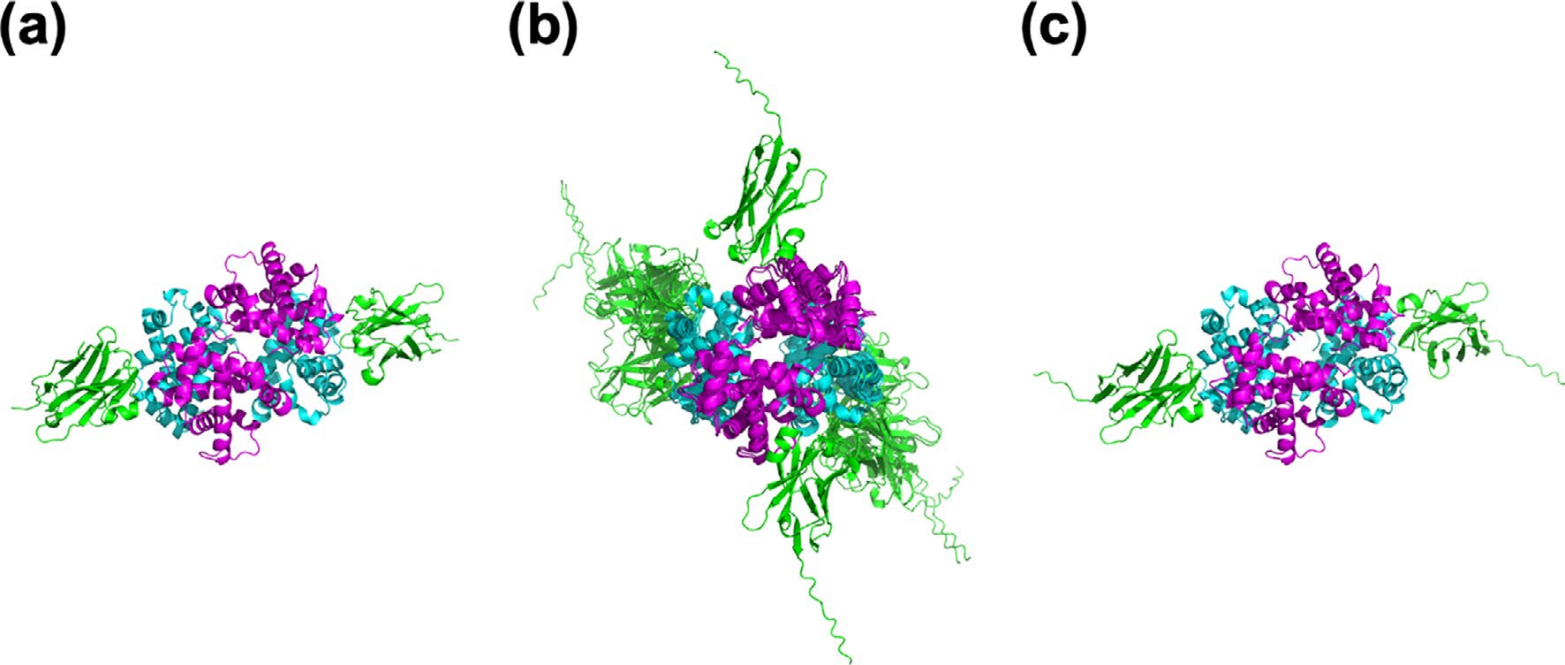
The submitted models for H1204. Hemoglobin chains are shown in cyan and magenta and two nanobody structures are in green. (a) The native structure (PDB ID: 8VYL) (b) Our 5 submitted models. The best DockQ: 0.490; RMSD: 13.8 Å. (c) The best DockQ model from KozakovVajda (H1204TS274_1). DockQ: 0.847; RMSD: 2.6 Å.

Target H1204, a complex between human hemoglobin (A2B2) and two nanobody units (C2), was an example of such a challenging case. While the structure of hemoglobin itself is well characterized with abundant template information, the primary difficulty lay in predicting the correct binding site and orientation of the nanobodies (Figure 9a). Antibody–antigen docking, including interactions with nanobodies, is generally considered a difficult task for methods like AF2 that heavily rely on co‐evolutionary signals, as these signals are often absent across binding interfaces between antigen and antibody. Reflecting this difficulty, many participating groups, including ours, failed to correctly identify the nanobody docking pose in their top‐ranked submissions. Consequently, our best submitted model for H1204 (Figure 9c) only achieved a DockQ of 0.490. The superimposition of all our submitted models, shown in Figure 9b, highlights the challenge in consistently predicting the correct nanobody placement. This indirectly suggests that methods like AF2 and AF3 may lack confidence and consistency in docking pose predictions when co‐evolutionary information is insufficient in the MSA.

In analyzing our results for this target, the primary limitation appears to lie not in the generation of accurate structural models, but in our scoring and ranking procedure. Post‐CASP analysis of our generated structure pool revealed that the model with the best DockQ in our pool had a DockQ of 0.552 and an RMSD of 5.6 Å. Submission of this model would have placed it 27th among all predictions for H1204 in the DockQ ranking. However, this high‐quality model was ranked extremely low by our scoring method, at the 169th position within our own pool, and thus was not selected for submission. The difficulties encountered with H1204 underscore that the accurate prediction and, critically, the reliable scoring and ranking of antibody/nanobody binding modes remain significant hurdles in computational structure prediction. The overall best prediction for H1204, submitted by the KozakovVajda group, achieved an impressive DockQ of 0.847, demonstrating that accurate generation of models and selecting such models were possible.

RNA structure prediction still faces fundamental limitations, especially for large or complex RNAs. While recent deep learning (DL) approaches have improved the accuracy of monomeric RNA structure prediction, their performance remains unsatisfactory compared to protein structure prediction. Moreover, RNA complex prediction was a major challenge in CASP16. For nearly all RNA multimer targets, no group, including ours, was able to produce a meaningful model. This is due in part to the extreme size and complexity of the RNA assemblies for the targets in this round. These results underscore the need for significant future developments in RNA multimer modeling.

## 4 Conclusion

We discussed our human group's performance in protein multimer and RNA structure prediction in CASP16. For the multimer targets, as probably most of the participating groups did, our predictions were based mainly on AF2 and AF3. However, our additional efforts, the use of Logan metagenomes, VoroIF‐jury score ranking, and literature information, contributed to our success on some targets, which led us to the top ranking among the participating groups in this category. Scoring and ranking strategies, while generally effective in our case, still have room for improvement. Also, large complexes, which do not fit into AF3 or other deep learning (DL)‐based methods, require further methodological development.

RNA prediction needs substantial development by the community. Long RNAs, RNA complexes, protein‐RNA, and RNA‐ligand complexes are in general not well modeled by AF3 or even cannot be handled by existing methods. A major limiting factor for DL‐based prediction methods is the lack of high‐quality training data, which constrains the effectiveness of data‐driven models. Addressing these challenges requires fundamental advancements, such as novel model architectures and the integration of diverse data modalities.

Finally, in real‐world biological applications, success will depend not only on the development of new models but also on the ability to efficiently utilize existing ones according to the specific properties of the target. Our approach relied not only on fully automated methods but also on expert intervention and literature information, which were successful for some targets. Although mining and using literature information are currently performed manually, as large language models (LLMs) and agent‐based artificial intelligence (AI) systems continue to advance, in the near future such tasks could be handled automatically by an AI‐based scientific reasoning and decision‐making pipeline.

## Author Contributions

**Yuki Kagaya:** software, methodology, investigation, validation, data curation, supervision, visualization, writing – original draft. **Tsukasa Nakamura:** methodology, software. **Jacob Verburgt:** methodology, investigation, validation, visualization. **Anika Jain:** investigation, validation, visualization, methodology. **Genki Terashi:** investigation, validation, methodology, visualization. **Pranav Punuru:** investigation, visualization, validation. **Emilia Tugolukova:** investigation, validation, visualization. **Joon Hong Park:** investigation, validation, visualization. **Anouka Saha:** investigation, validation, visualization. **David Huang:** investigation, validation. **Daisuke Kihara:** conceptualization, methodology, validation, funding acquisition, writing – review and editing, project administration, resources.

## Conflicts of Interest

The authors declare no conflicts of interest.

## Data Availability

The data that support the findings of this study are available in CASP16 archive at https://predictioncenter.org/download_area/CASP16/. These data were derived from the following resources available in the public domain: PDB, https://www.pdbj.org.
